# Supplementary material for: Integrating Explicit and Implicit Fullerene Models into UNRES Force Field for Protein Interaction Studies
Source: Molecules. 2024 Apr 23;29(9):1919. doi: 10.3390/molecules29091919 (PMC11085604; doi:10.3390/molecules29091919)
Supplement: Supplementary file 1 [file molecules-29-01919-s001.zip › molecules-2958823-supplementary.pdf]

Supplementary Materials for

Integrating Explicit and Implicit Fullerene Models into UNRES Force Field for Protein Interaction Studies

Natalia Rogoża, Magdalena A. Krupa, Pawel Krupa* and Adam K. Sieradzan
*Corresponding author. Email: pkrupa@ifpan.edu.pl

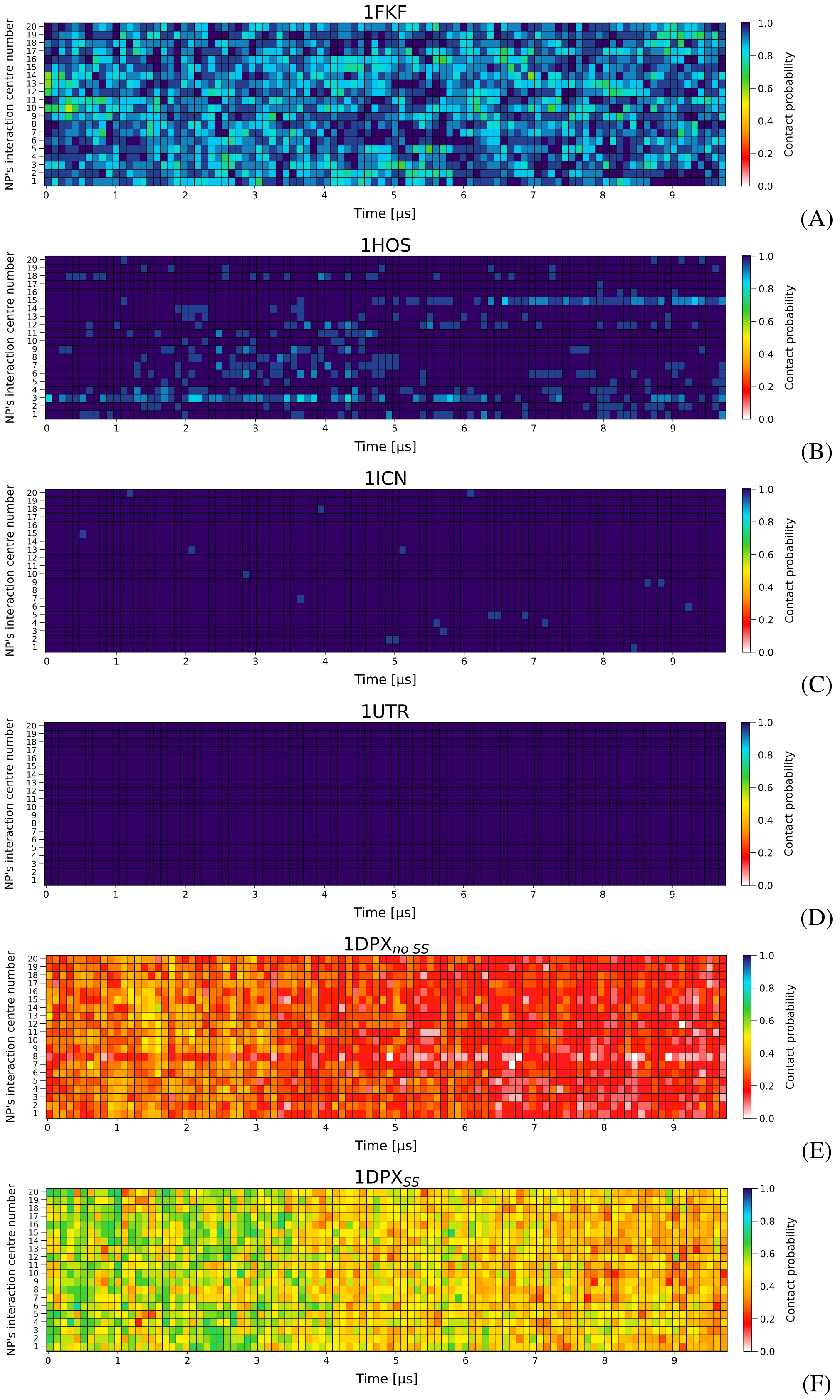

Figure S1: Contact probability of nanoparticle interaction centers as a function of time averaged over 20 trajectories for: (A) 1FKF, (B) 1HOS, (C) 1ICN , (D) 1UTR, (E) 1DPX$_{no\ SS}$, and (F) 1DPX$_{SS}$ protein performed with UNRES force field in simulations with explicitly defined fullerene nanoparticle.

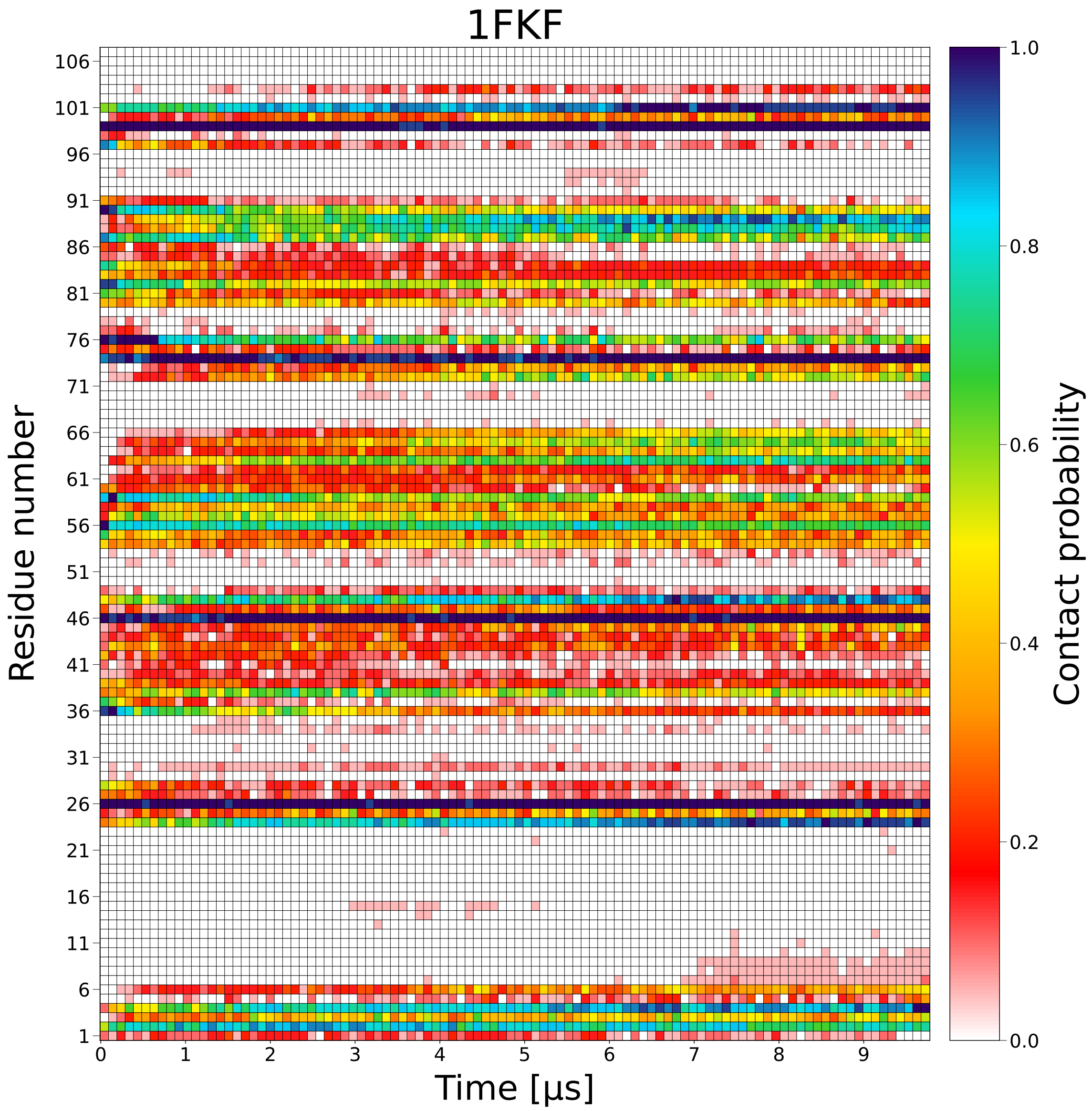

1FKF
Residue number
Time [μs]
Contact probability
106
101
96
91
86
81
76
71
66
61
56
51
46
41
36
31
26
21
16
11
6
1
0
1
2
3
4
5
6
7
8
9
1.0
0.8
0.6
0.4
0.2
0.0

(A)

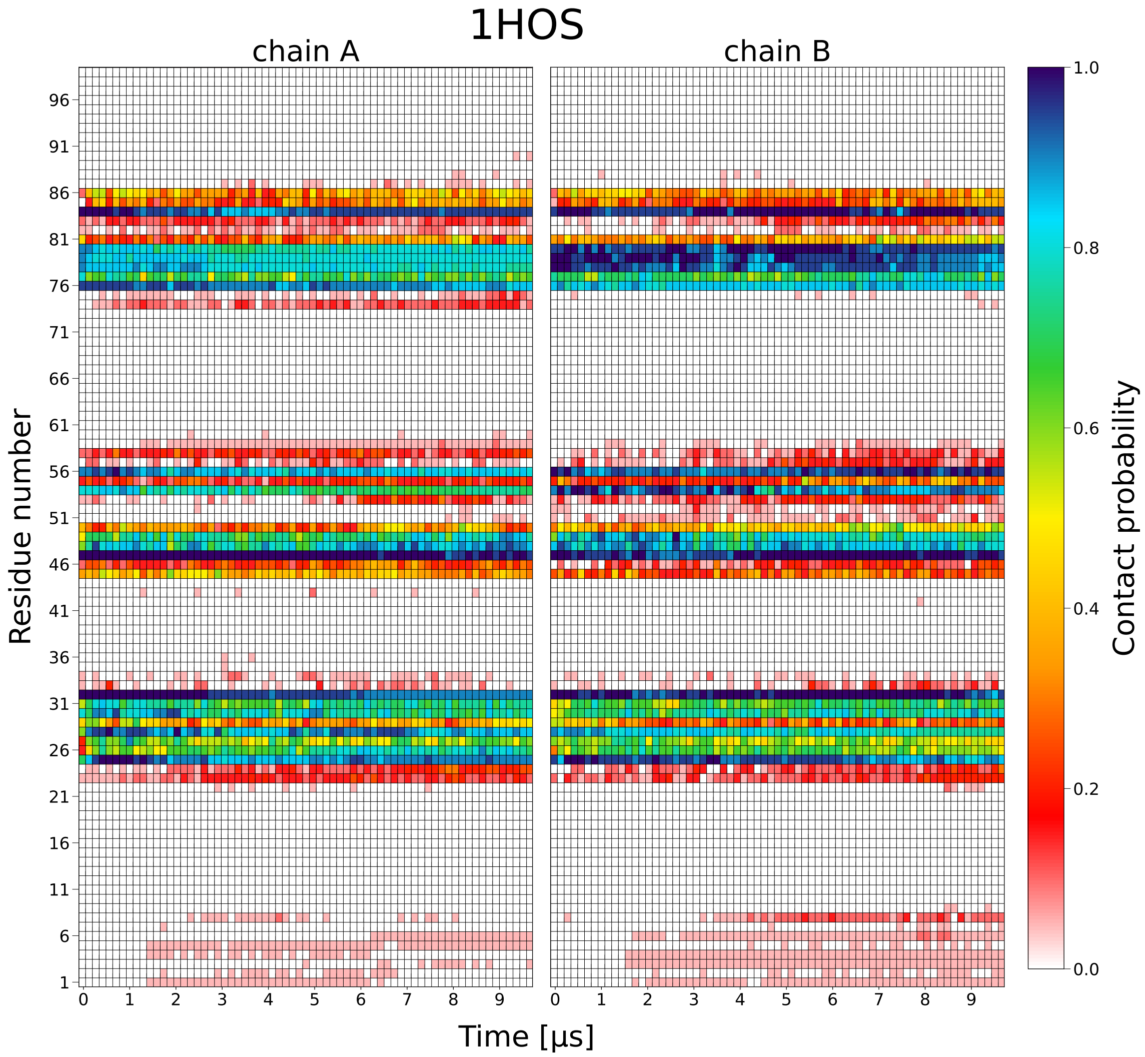

1HOS
chain A
chain B
Residue number
Time [μs]
Contact probability

(B)

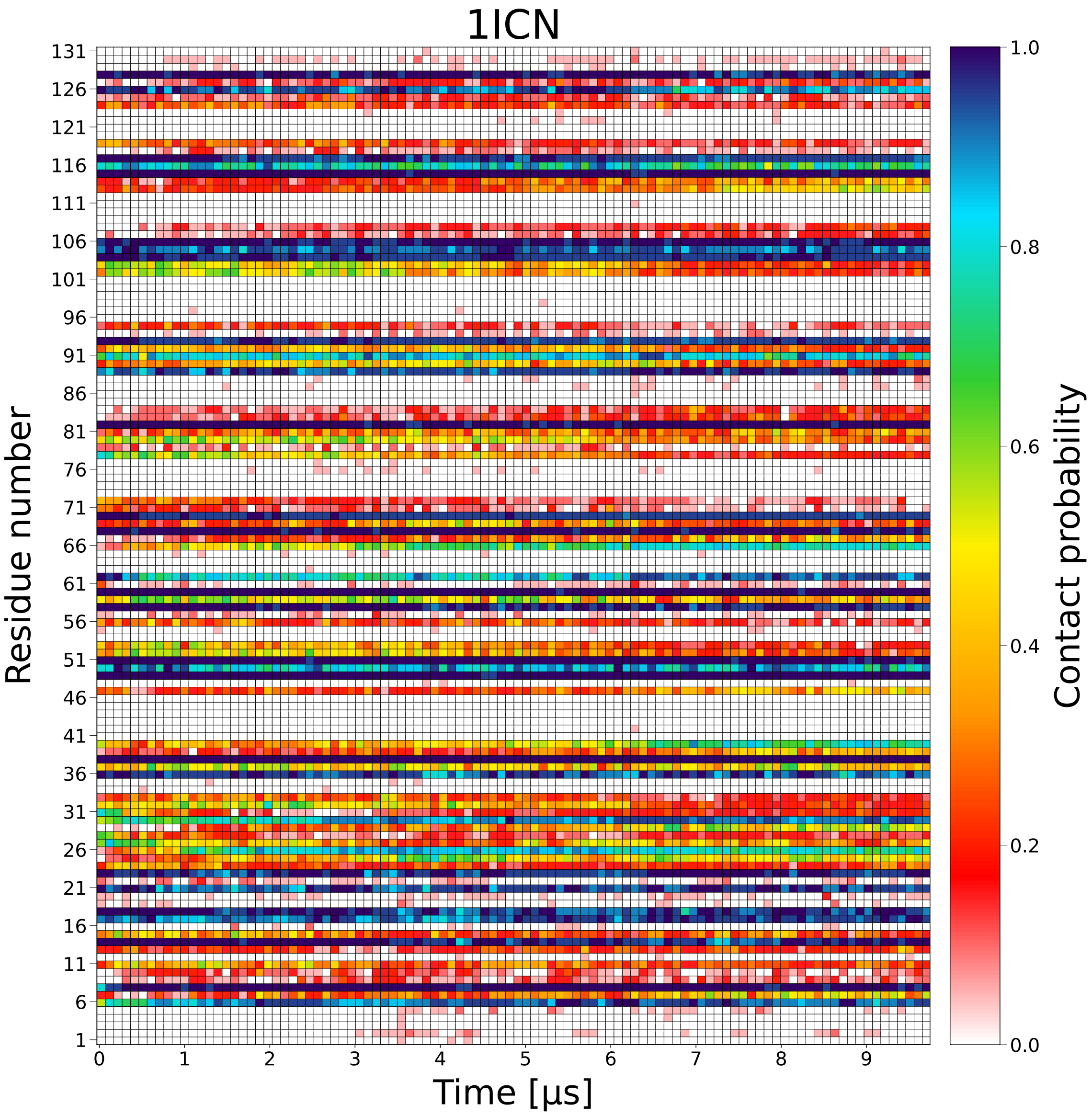
1ICN
131
126
121
116
111
106
101
96
91
86
81
76
71
66
61
56
51
46
41
36
31
26
21
16
11
6
1
Residue number
0
1
2
3
4
5
6
7
8
9
Time [μs]
1.0
0.8
0.6
0.4
0.2
0.0
Contact probability

(C)

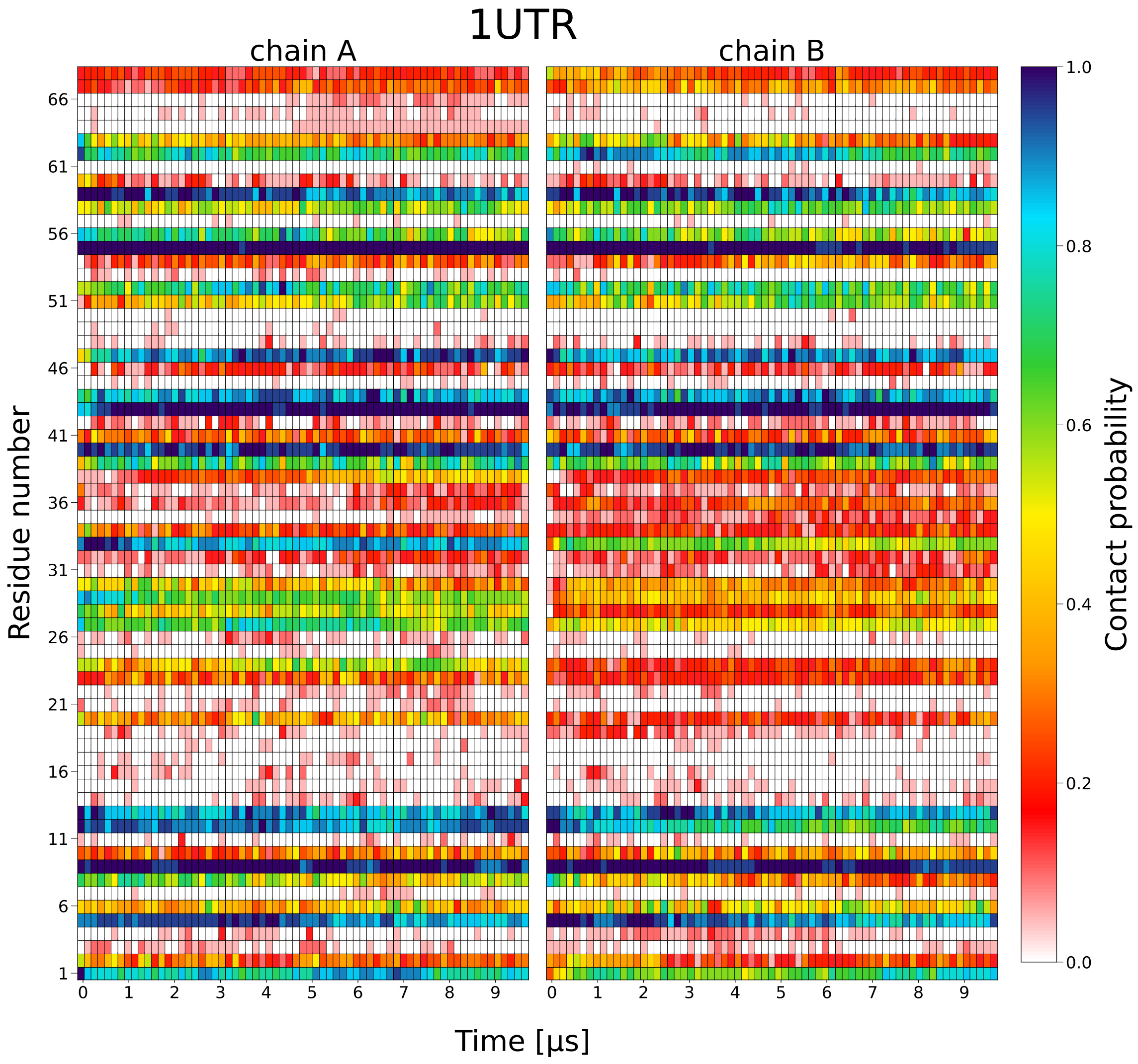
1UTR
chain A
chain B
Residue number
66
61
56
51
46
41
36
31
26
21
16
11
6
1
0
1
2
3
4
5
6
7
8
9
Time [μs]
Contact probability
1.0
0.8
0.6
0.4
0.2
0.0

(D)

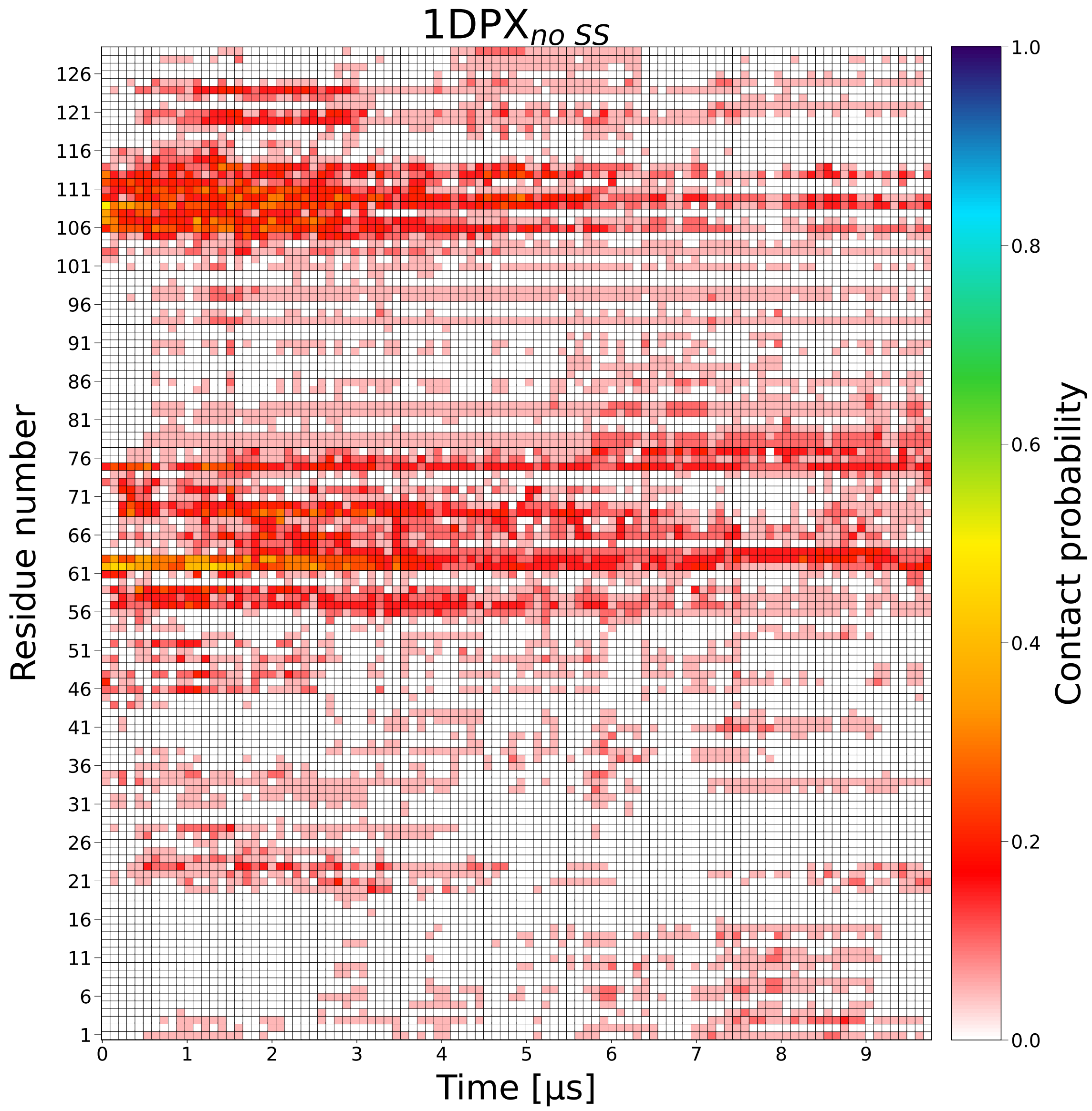

1DPX$_{no\ SS}$
Residue number
Time [μs]
Contact probability
126
121
116
111
106
101
96
91
86
81
76
71
66
61
56
51
46
41
36
31
26
21
16
11
6
1
0
1
2
3
4
5
6
7
8
9
1.0
0.8
0.6
0.4
0.2
0.0

(E)

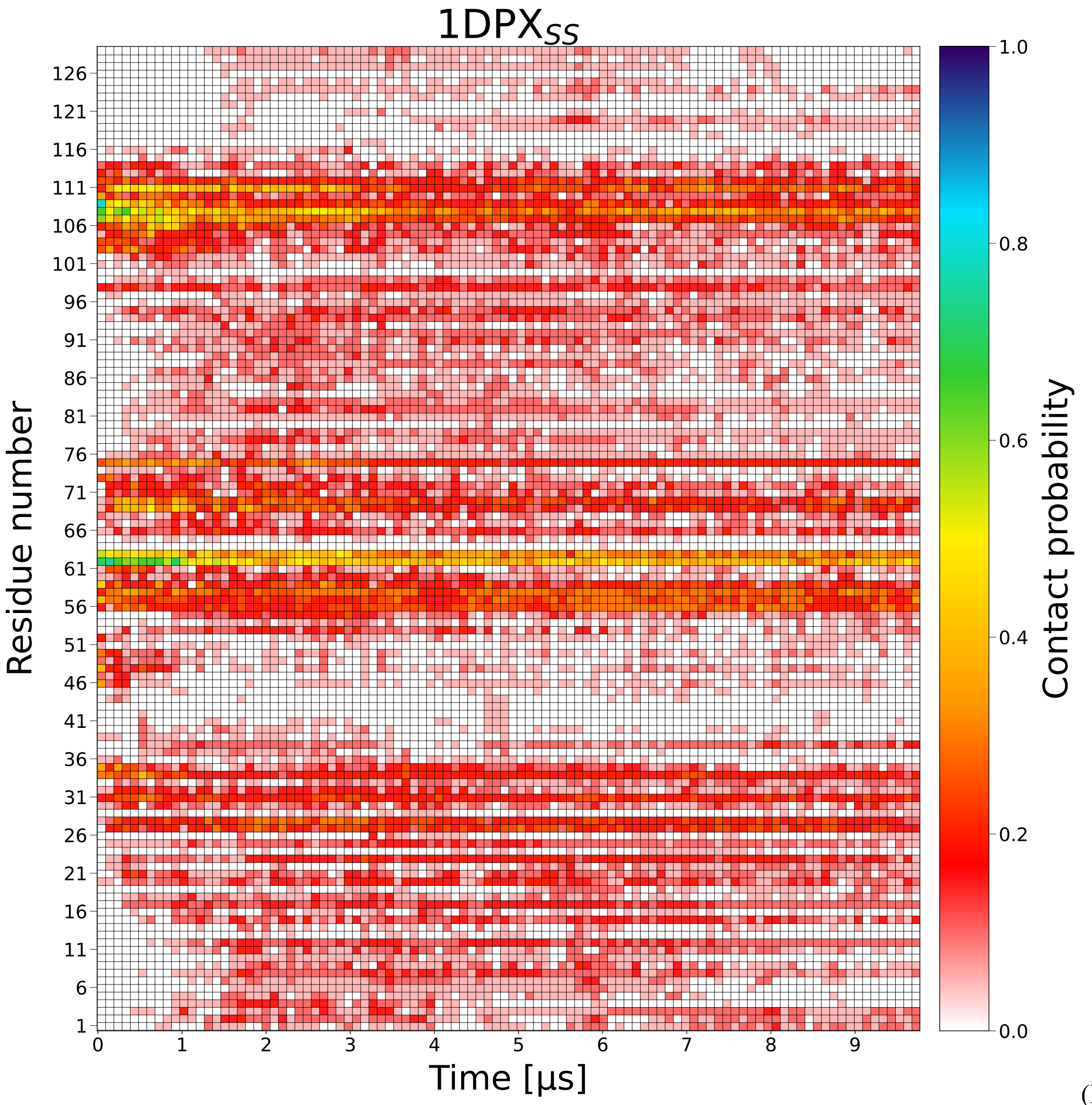

(F)

Figure S2: Contact probability of protein residues as a function of time averaged over 20 trajectories for (A) 1FKF, (B) 1HOS, (C) 1ICN, (D) 1UTR, (E) 1DPX$_{no\ SS}$, and (F) 1DPX$_{SS}$ protein performed with UNRES force field in simulations with explicitly defined fullerene nanoparticle.

Interaction center	Fullerene model					
	Implicit		Explicit			
	ϵ [kcal/mol]	σ [Å]	ϵ_0 [kcal/mol]	σ [Å]	σ_0 [Å]	χ_0‘
Peptide group	1.197	5.367	-1.500	4.000	*	*
Cys	1.553	5.644	5.264	2.675	4.927	0.870
Met	1.668	5.668	5.486	2.734	5.105	1.054
Phe	1.661	5.938	5.291	2.966	4.207	0.939
Ile	1.742	5.863	5.884	2.882	4.851	1.026
Leu	1.731	5.995	5.671	3.021	2.785	1.083
Val	1.632	5.832	5.551	2.841	3.583	1.054
Trp	1.535	5.496	4.757	2.477	7.866	0.789
Tyr	1.360	5.394	4.163	2.461	7.430	0.899
Ala	1.322	5.437	4.386	2.465	1.963	1.003
Gly	1.197	5.367	3.652	2.493	0.799	1.243
Thr	1.033	5.544	3.025	2.573	4.058	0.893
Ser	0.985	5.378	2.568	2.456	1.889	0.917
Gln	0.975	5.400	2.500	2.484	3.198	1.616
Asn	0.902	5.418	2.139	2.489	3.267	1.432
Glu	0.770	5.468	1.471	2.509	2.685	2.050
Asp	0.755	5.469	1.737	2.508	2.004	1.420
His	1.198	5.347	3.105	2.422	6.244	0.993
Arg	0.968	5.297	2.015	2.271	8.196	1.432
Lys	0.921	5.375	1.820	2.452	13.475	27.495
Pro	1.121	5.672	3.640	2.703	2.663	0.779

Table S1: ϵ and σ parameters used for Kihara potential for nanoparticle in implicit form and the Gay-Berne potential parameters ϵ and $\sigma 0_i$ (σ_i) of indidual residue i and χ‘, where $\sigma 0$, (σ) is distance corresponding to the 0 value of E$_{Gay-Berne}$ at side-to-side (head-to-head) orientation from which χ is computed. * peptide group interact through LJ potential, negative ϵ_0 value indicate that both 6 and 12 terms are repulsive

To compute σ (or $\sigma 0$) a transfromation is applied $sigma_{ij} = \sqrt{sigma_i^2 + sigma_j^2}$. Therefore, for nanoparicle $sigma_{nano,j} = \sqrt{sigma_{Ala}^2 + sigma_j^2}$. To transform χ_0‘ to χ‘ a given equation is used:

$$\chi‘ = (\chi_0‘ - 1)/(\chi_0‘ + 1)$$

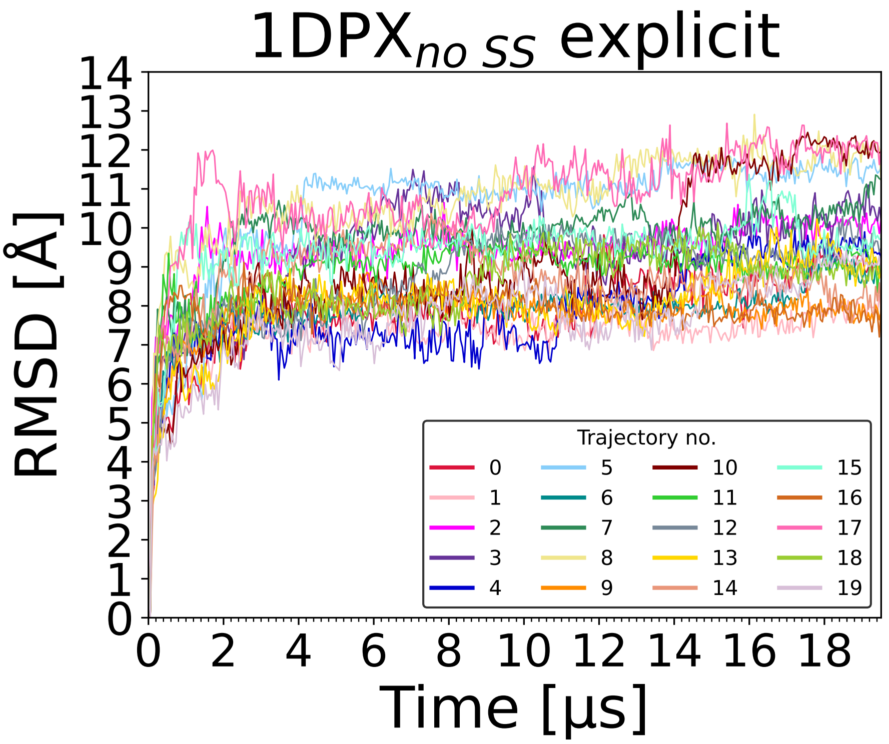

(A)

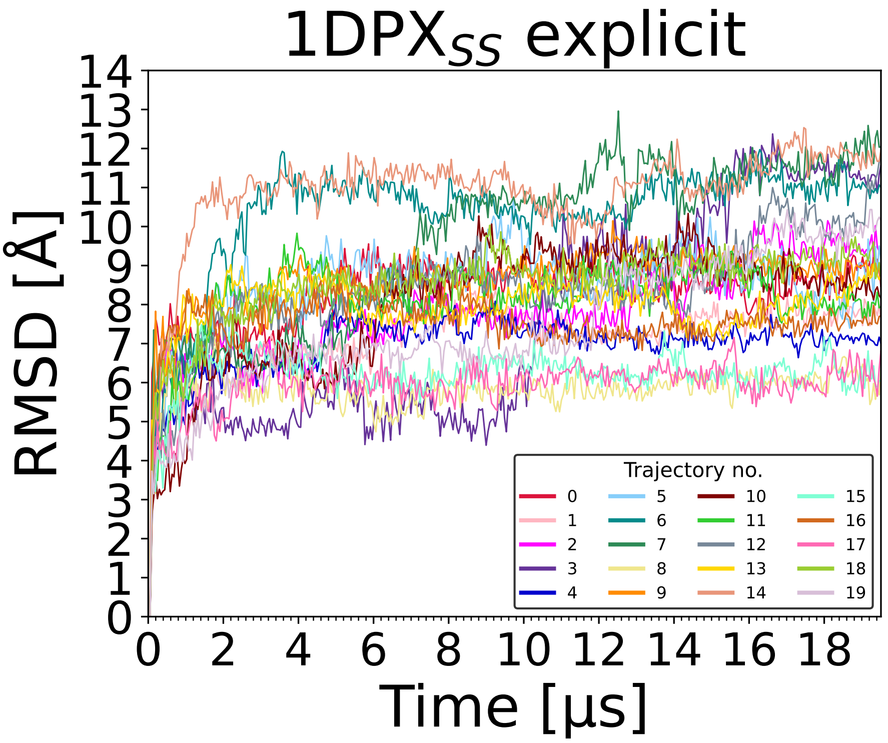

(B)

Figure S3: RMSD values over time for each of 20 trajectory in explicit simulations for (A) 1DPX$_{no\ SS}$ and (B) 1DPX$_{SS}$ protein performed with UNRES force field.
